# Distinguishing Direct Human‐Driven Effects on the Global Terrestrial Water Cycle

**DOI:** 10.1029/2022EF002848

**Published:** 2022-08-18

**Authors:** Elisie Kåresdotter, Georgia Destouni, Navid Ghajarnia, Richard B. Lammers, Zahra Kalantari

**Affiliations:** ^1^ Department of Physical Geography and Bolin Centre for Climate Research Stockholm University Stockholm Sweden; ^2^ Earth Systems Research Center Institute for the Study of Earth, Oceans, and Space University of New Hampshire Durham NH USA; ^3^ Department of Sustainable Development Environmental Science and Engineering (SEED) KTH Royal Institute of Technology Stockholm Sweden

**Keywords:** global hydrological modeling, human‐water interactions, anthropogenic hydrological change, runoff, evapotranspiration, storage change

## Abstract

Population growth is increasing the pressure on water resource availability. For useful assessment and planning for societal water availability impacts, it is imperative to disentangle the direct influences of human activities in the landscape from external climate‐driven influences on water flows and their variation and change. In this study we used the water balance model, a gridded global hydrological model, to quantify and distinguish human‐driven change components, modified by interventions such as dams, reservoirs, and water withdrawals for irrigation, industry, and households, from climate‐driven change components on four key water balance variables in the terrestrial hydrological system (evapotranspiration, runoff, soil moisture, storage change). We also analyzed emergent effect patterns in and across different parts of the world, facilitating exploration of spatial variability and regional patterns on multiple spatial scales, from pixel to global, including previously uninvestigated parts of the world. Our results show that human activities drive changes in all hydrological variables, with different magnitudes and directions depending on geographical location. The differences between model scenarios with and without human activities were largest in regions with the highest population densities. In such regions, which also have relatively large numbers of dams for irrigation, water largely tends to be removed from storage and go to feed increased runoff and evapotranspiration fluxes. Our analysis considers a more complete set of hydrological variables than previous studies and can guide further research and management planning for future hydrological and water availability trends, including in relatively data‐poor parts of the world.

## Key Points

- Human activities affected all four hydrological variables studied (runoff, evapotranspiration, soil moisture, and storage change)
- Human‐driven hydrological effects emerged as considerable over the northern tropical to temperate latitudes, where most people live
- Higher runoff was seen in areas with dams of various types, with additional hydrological effects depending on dam type

## 1 Introduction

Population growth is increasing human demands and pressures on water resources, for example, by changing land use and cover and redirecting water for different types of uses, such as agricultural, urban, and industrial, with water availability effects that may even exceed those of climate change (Bierkens, 2015; Destouni et al., 2013; Haddeland et al., 2013; Vörösmarty et al., 2000). In parallel, climate change is altering spatial‐temporal precipitation patterns around the globe, further affecting water flow regimes and potentially compromising water security (Eekhout et al., 2018; Hewitson et al., 2014; Zeng et al., 2017). As climate change and human activities can alter hydrological conditions in different ways, it is essential to distinguish their respective effects on different hydrological variables. Understanding the hydrological variability and change responses to different anthropogenic pressure changes is important for several reasons, including to decipher past and predict future trends, relate them to potential societal water conflicts, and develop sustainable water management strategies. However, observational data are frequently lacking, creating a need for modeling to gain insights into such cause‐effect relationships. Historical observational hydro‐climatic data, for example, on precipitation and runoff, are also not uniformly distributed around the world, with both data availability and data resolution depending on geographical location, and some areas completely lacking historical data (Döll et al., 2016; Wada et al., 2017). Model‐based experimentation can enable global investigation of hydrological responses to human activity developments, including in regions that lack historical data.

In general, studies of hydrological and water availability change trends need to consider their connections with societal processes and human activities (Jing Liu et al., 2017; Konar et al., 2019; Wu et al., 2017; Zaveri et al., 2016). Many data‐driven and model‐based studies have addressed how human activities affect water flows and availability on different scales. These include local catchment studies (Bissenbayeva et al., 2019; Fenta et al., 2017; Jiajia Liu et al., 2019; Khandu et al., 2016; Khazaei et al., 2019; Li et al., 2020; Rakhimova et al., 2020; Wu et al., 2017) and multi‐catchment studies up to country or larger scale (Destouni et al., 2013; Dey & Mishra, 2017; Grogan et al., 2015; Haddeland et al., 2013; Moshir Panahi et al., 2020; Zuidema et al., 2020). Global studies often investigate one or two selected hydrological variables, such as runoff or evapotranspiration (Felfelani et al., 2017; Gordon et al., 2005; Jaramillo & Destouni, 2014, 2015; Liu et al., 2021; Müller Schmied et al., 2016; Rost et al., 2008; Veldkamp et al., 2017; Wada et al., 2016), or some specific hydrological subsystem such as groundwater, or aspect such as water scarcity (Grogan et al., 2017; Hanasaki et al., 2018; Vörösmarty et al., 2000; Zeng et al., 2017). Global hydrological models have been developed to represent more fully how human activities may affect water flows and availability, but parameterizations differ between models and large‐scale human‐water interactions still remain insufficiently well understood, perhaps even less so than climate‐water interactions (Bierkens, 2015; Telteu et al., 2021). To consistently understand socio‐hydrological connections, multiple hydrological variables and subsystems need to be considered in order to capture and decipher their hydrological inter‐linkages (Ghajarnia et al., 2021; Orth, 2018), in combination with their interactions with human activities and societal water aspects. Moreover, different scales and parts of the world need to be accounted for, since human‐water system interactions work simultaneously across these, for example, by virtual water use with water embedded in traded goods (Gu et al., 2021; Konar et al., 2019; Wu et al., 2017) and energy system choices (Engström et al., 2021).

To address these research needs and contribute to bridging current gaps, this study aimed to identify human effects on multiple key water balance variables of the hydrological system (evapotranspiration, runoff, soil moisture, storage change), and emergent effect patterns in and across different parts of the world. To achieve this aim, we used the Water Balance Model (WBM), a global gridded hydrological model (Grogan et al., 2022). This enabled exploration of spatial variability and regional patterns in the interactions of human activities with hydrology on multiple spatial scales, from pixel up to global, including in previously uninvestigated parts of the world.

## 2 Materials and Methods

The global gridded WBM (Grogan, 2016; Grogan et al., 2022; Vörösmarty et al., 1989; Wisser et al., 2010) simulates water flow at daily time steps, both as vertical water exchange between the land surface and atmosphere (quantifying evapotranspiration) and as horizontal water transport (quantifying runoff and its area integration into stream/river discharge), with spatial resolution of 0.5°. The model simulates the direction of hydrological flows and the effect that different human activities (e.g., dams) and climate change (e.g., precipitation changes) have on hydrological outcomes. These outcomes consist of historical time series of different hydrological variables (evapotranspiration, runoff, soil moisture, storage change). Further model details and documentation can be found in the original sources (Grogan, 2016; Grogan et al., 2022; Wisser et al., 2010).

**Figure 1:**
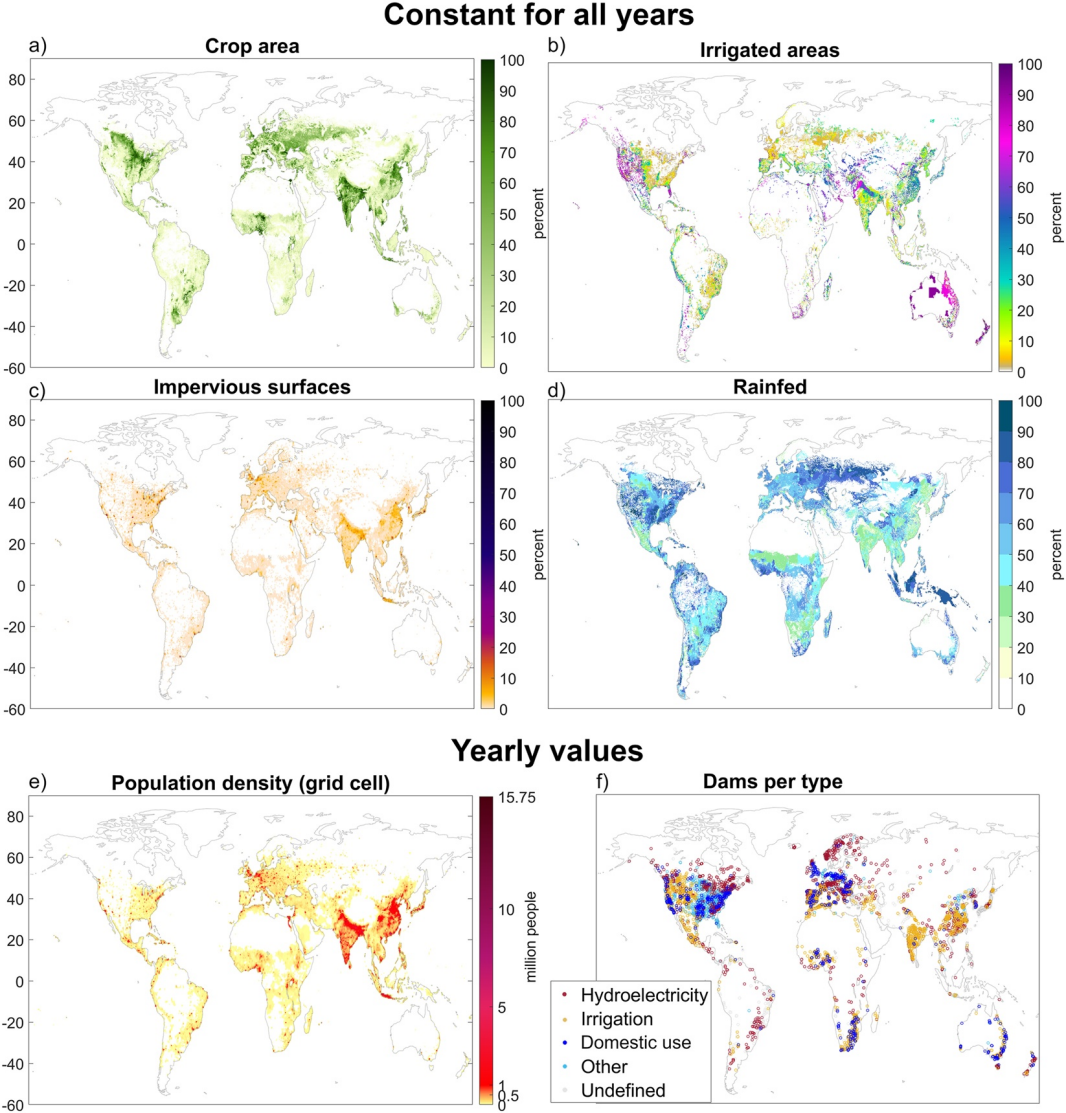
Important human components considered in the “human impact” (HI) simulation scenario with the water balance model (WBM). Crop area (a), irrigated areas (b), impervious surface (c) and rainfed areas (d) are constant for all years in the model, while population density (e) and dams (f) have yearly values. Population density shown is the 70‐year average, and dams show all dams in the last year.

**Figure 2:**
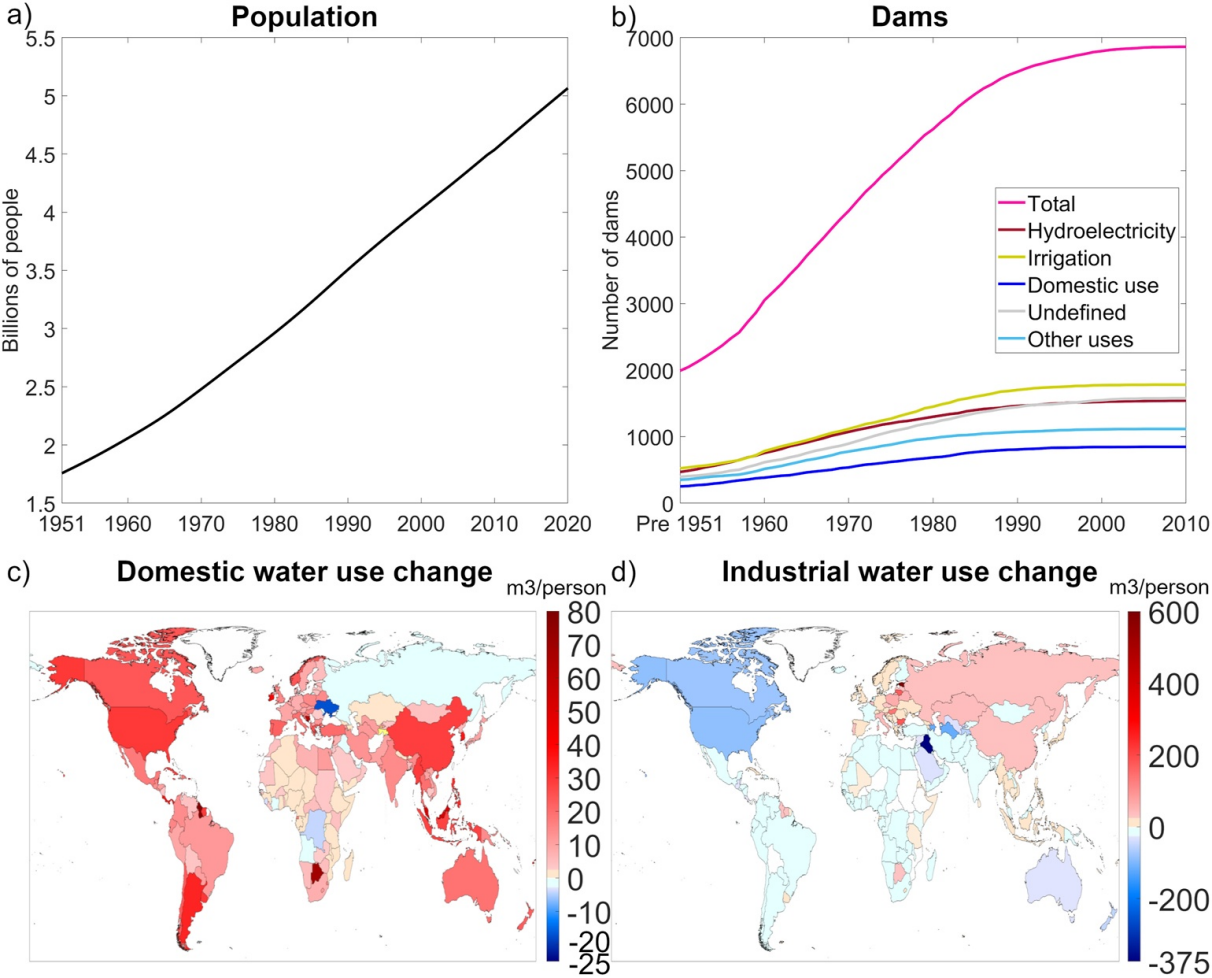
Yearly change in (a) population for the investigated area, (b) accumulated number of dams per type, and long‐term per capita changes in water use per country between 1981 and 2020 for the domestic (c) and industry (d) sectors (increased use: red nuances, decreased use: blue nuances, no change: white).

In this study, we followed a similar approach to that used in previous model experimentation studies seeking to distinguish human‐driven impacts on resulting hydrological variables (Felfelani et al., 2017; Müller Schmied et al., 2021; Veldkamp et al., 2017; Wada et al., 2016). Specifically, we created two different simulation scenarios, with the first “human impact (HI)” scenario including consideration of different human activities, such as irrigation, impervious surfaces in urban areas, and dams, and the second “no human impact (NHI)” scenario excluding all human activity modules in the model and considering only natural processes. Thus, the NHI scenario simulated what hydrological flows would look like if no humans were present to alter the water cycle, while the HI scenario was a representation of actual reality with humans in place. For both scenarios, outputs were generated worldwide, with spatial resolution of 0.5°, for all hydrological variables (runoff, evapotranspiration, soil moisture, storage change) and with the same climate boundary conditions (precipitation, air temperature) on a daily basis for the 70‐year period 1951–2020. For both WBM simulation scenarios, we utilized the data set on climate variables provided by the European Centre for Medium‐Range Weather Forecasts (ECMWF) Reanalysis fifth Generation (ERA5) (Bell et al., 2020; Hersbach et al., 2018), thus keeping climate conditions, as represented by precipitation and air temperature variables, the same in both the HI and the NHI scenarios. To simulate the impacts of human activities on the selected hydrological variables, using available model modules we considered eight human components: inter‐basin transfers, dams, reservoirs, impervious surfaces, domestic and industrial water demand, livestock water demand, irrigated agriculture, and unsustainable water use (water “mining”). Some of these are shown in Figure 1. Some human activities, such as crop area, animals per capita, domestic and industrial water use per capita, impervious surfaces, irrigation efficiency, and irrigated area, were held constant in the WBM simulations for all years. Other human activities, such as population density, number and location of dams (up until 2010), and domestic and industry water use were varied from year to year, with global change over time shown in Figure 2. Yearly water use per sector uses Liu et al. (2017), where water use was calculated for the years 1981 onward, with previous years filled using the values for the year 1981. Dams with main use defined as recreation, fisheries, navigation, flood control, and other constituted approximately 6% of total dams and were grouped together, forming a category called “other uses.” As Figure 1 shows, world regions with the highest population density also tend to have the highest level of human activities, such as dams, agriculture, and impervious surfaces. Population growth over time appears to be linear (Figure 2a), but with fewer dams built from 1990 onward (Figure 2b). The GRandDam (Lehner et al., 2011) database version 1.1 was utilized in the model, which could explain the trend for less dams being built toward the end of the simulations (Figure 2). The updated 1.3 version contains an additional 458 dams, with almost all of these being constructed from 1990 onward. With the inclusion of more dams, the resulting differences between the scenarios could potentially have been even greater. The glacier module in WBM was not used in the simulations, and all areas with permanent ice sheets and glaciers on land (RGI Consortium, 2017), including all of Greenland and the Antarctic, were therefore masked in the model output. Extreme result outliers were excluded from the analysis and only results between the 1st and 99th percentile was considered.

To evaluate result differences between the HI and NHI scenarios, we extracted the daily values for each hydrological variable and grid cell and calculated their long‐term average values over the full 70‐year simulation period. We then subtracted the long‐term average for each variable and pixel in the NHI scenario from that in the HI scenario. By mapping the resulting difference between the two scenarios for each variable, we were able to analyze human effects on the selected hydrological variables and emergent effect patterns around the globe. As the model results showed the cumulative impact of all human activities on the hydrological variable, the contribution of each activity could not be analyzed. Instead, our analysis focused on finding spatial connections between large scenario differences and the direction and magnitude of different human activities. The student's *t*‐test was utilized to calculate grid cells with significant resulting differences (*p* ≤ 0.05) between the two scenarios for each variable. To further analyze regional effect patterns, we split the yearly and long‐term scenario results and their differences into 10‐degree latitude bands, for which we also calculated and mapped corresponding mean latitudinal values. To allow direct population comparison between the latitude bands, irrespective of their variable land area (number of grid cells), we recalculated population density from per square kilometer to population per grid cell. For dams the numbers were aggregated, resulting in number of dams per dam type and 10‐degree latitude interval. Mean population per grid cell and total number of dams per type at year 2020 were also split into 10‐degree latitude bands for comparison with scenario results. Spearman correlation and corresponding *p*‐values were calculated for 10‐degree latitudinal bands using yearly values of all hydrological differences, population, and dams (total per type), to further investigate potential relationships between changes in hydrological variables and human activities. In Figure 2, population is shown only for areas included in the analysis, and thus the values are lower than total global population.

## 3 Results and Discussion

### 3.1 Human Effects on Key Hydrological Variables

**Figure 3:**
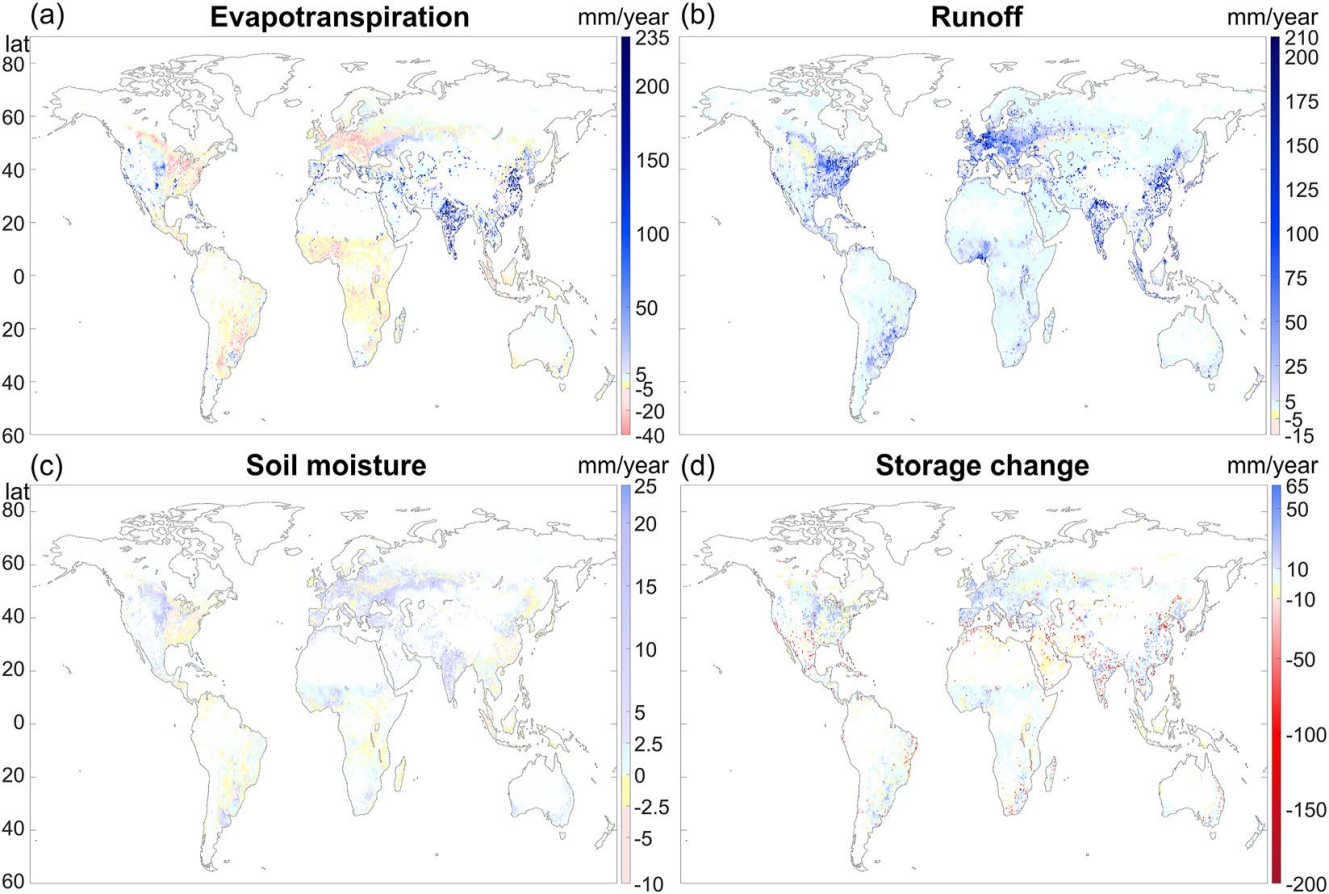
Result differences between simulations in the human impact (HI) and no human impact (NHI) scenarios for (a) evapotranspiration, (b) runoff, (c) soil moisture, and (d) storage change. The differences quantify the effects of human activities on the long‐term average values of the hydrological variables (increase: blue nuances; decrease: red nuances, differences close to or equal to zero: white). The results considered are within the 1st to 99th percentile interval to avoid extreme result outliers.

Comparison of the WBM results for the HI and NHI scenarios revealed large spatial variation in the scenario differences for all four hydrological variables studied (Figure 3). All areas were analyzed for connections in result differences between simulations in the scenarios and all different human activities. Population and dams were found to be associated with large scenario differences, while other human activities showed no significant impact on the differences between the HI and NHI scenarios. As can be expected for human activity effects, areas with relatively high population density (Figure 1a) tended to show relatively large scenario differences, that is, considerable apparent effects of human interventions on the hydrological variables studied. In the most densely populated areas in Asia, evapotranspiration (Figure 3a), runoff (Figure 3b), and soil moisture (Figure 3c) tended to be mostly greater in the HI than NHI scenario, while storage change tended to be mostly smaller (i.e., storage decrease was greater or storage increase was smaller, Figure 3d). In some parts of Europe, North and South America, and Africa, evapotranspiration was lower and storage change was slightly greater (i.e., greater increase or smaller decrease), while runoff was also greater in the HI than in the NHI scenario. For soil moisture, lower values emerged in the HI than the NHI scenario for North America, Eastern Europe, along the east coast of China, Japan, and throughout the equatorial regions (Figure 3c), where extensive impervious surfaces tend to be concentrated (Figure 1c). This is consistent with such surfaces lowering water infiltration into the soil and increasing surface runoff (Shuster et al., 2005). Consistently for areas with relatively low population density and degree of human activities (Figure 1), human effects on hydrology emerged as relatively small, with mean and median values of scenario result differences close to zero (white grid cells, Figure 3). There was a strong to very strong positive correlation in the yearly values for difference between simulations of runoff and population. In other words, runoff tends to follow population. A very strong negative correlation was found between difference in storage and population for these areas with a higher number of dams. Interestingly, for other latitudes (65°N, 75°N, 45°S), a strong to very strong positive correlation emerged instead. It should be noted, however, that the latter are among the least populated areas worldwide. For the most populated areas (latitudes 15°–45°N) a strong to very strong positive correlation was found for evapotranspiration and population.

**Figure 4:**
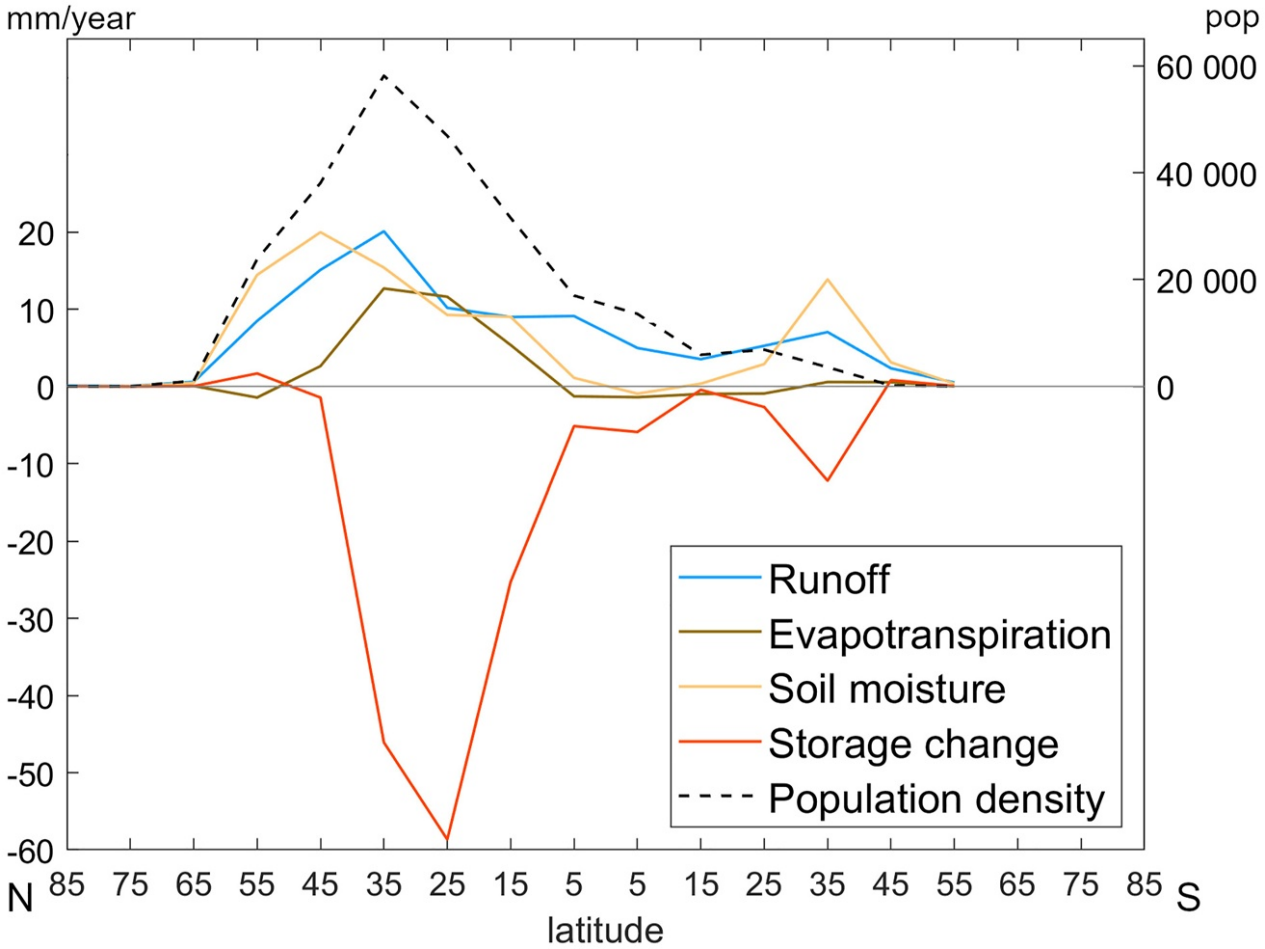
Scenario differences in long‐term average values of hydrological variables (in the 1st to 99th percentile interval) and population density (individuals per grid cell) over 10‐degree latitude bands with results plotted in mid‐band locations.

Close to all land surface grid cells showed significant result differences, however, areas with low soil moisture values in the HI scenario, such as the Sahara Desert and central parts of Australia, and areas with little to no soil moisture differences between the scenarios, such as large parts of Canada and Scandinavia, showed less significant result differences. In areas where soil moisture is close to zero, percentage differences from year to year were more drastic, which could explain why these areas showed less significant changes than other areas. Figure 4 shows large‐scale spatial patterns obtained by aggregating scenario differences over 10‐degree latitude bands around the world. This confirmed the expected result of human intervention effects on hydrological variables tending to follow population density, with the largest differences between the HI and NHI scenarios emerging over the northern tropical to temperate regions that have the highest human population density. Over these regions with considerable human effects, runoff, evapotranspiration, and soil moisture tended to be greater in the HI than the NHI scenario, while water storage change tends to be smaller (less increasing or more decreasing).

However, these scenario differences may to some degree have been forced by assuming the same climate in both scenarios, as commonly done in model experimentation studies seeking to distinguish human impacts on terrestrial hydrology (Felfelani et al., 2017; Müller Schmied et al., 2021; Veldkamp et al., 2017; Wada et al., 2016). Specifically, with human withdrawals and consumptive use of water from storage considered in the HI, but not the NHI scenario, cumulative storage change (increase or decrease) must overall tend to be smaller in the HI than in the NHI scenario, in particular for areas with a large population withdrawing and consuming water. The basic water balance then requires modeled evapotranspiration and runoff to be greater in those areas, since less of the same available precipitation water input goes to feed the smaller water storage in the HI than in the NHI scenario. In reality, however, climate change co‐occurs with, and is to some degree also driven by, how humans use land and water, creating more complex regional climate‐water interactions, feedbacks, and impacts over time. For example, the considerable evapotranspiration differences emerging from the comparative scenario modeling itself for some regions (Figure 4), challenge the underlying assumption of the same climate in both scenarios, as they imply different climate‐regulating latent heat flux and thus different local‐regional climate implications (Destouni et al., 2010) for the hypothetical NHI than the realistic HI scenario. Moreover, human land‐ and water‐use developments (e.g., irrigation expansion) that were held constant in the model may also have combined societal and climate drivers (e.g., need for food supply enhancement and precipitation decrease, respectively) that feed back to each other and also alter hydrological responses and feedbacks to climate change (Jarsjö et al., 2012). In contrast to the largest regional scenario differences in Figure 4, where both evapotranspiration and runoff increased with the additional water provided exclusively from storage depletion (since precipitation was assumed the same in the NHI as in the HI scenario), observation data for large areas around the world often show opposite change directions in runoff and evapotranspiration under combined precipitation change (in any direction) and storage depletion (Destouni et al., 2010; Jaramillo & Destouni, 2014; Karlsson et al., 2015; Moshir Panahi et al., 2020). Some caution is therefore needed in interpreting comparative model results for human activity impacts on hydrology with the same climate assumed in different activity scenarios.

Previous data‐driven studies of human land and water use developments in parallel with climate change over time have shown various hydrological effects. For example, Khazaei et al. (2019) found human‐driven runoff decrease, while Liu et al. (2019) found human‐driven evapotranspiration decrease. Other data‐driven studies have found opposite flux change directions, that is, increases in evapotranspiration and decreases in runoff, or vice versa, depending on the specific combinations of human land and water uses and precipitation changes co‐occurring over each respective time period and location of study (Destouni et al., 2013; Jaramillo & Destouni, 2014). Land‐cover based calculations by Gordon et al. (2005) indicated human‐driven effects of decreased evapotranspiration, for example, due to deforestation and excluding irrigated agriculture. This is in line with findings for several regions in the present study, such as the east coast of North America, Central Europe, and sub‐Saharan Africa (Figure 3a). Regions with many irrigation dams and active irrigation, such as large parts of southeast Asia and the American west coast, instead showed increased evapotranspiration (Figure 3a), in line with several previous findings for hydrological effects of irrigation (Asokan & Destouni, 2014; Destouni et al., 2013; Destouni & Prieto, 2018; Gordon et al., 2005; Jaramillo & Destouni, 2015). Areas with human‐driven storage changes found in this study are also largely consistent with human‐driven water storage anomalies reported by Liu et al. (2021) using Gravity Recovery and Climate Experiment data. A study by Zeng et al. (2017) found runoff and soil moisture increases in areas with high groundwater extraction and associated storage decreases, such as northern India and Pakistan, the north China plain, and the central United States. Comparison of results for these groundwater extraction hotspots showed similar directions of change (increase or decrease) for runoff and soil moisture, and also similar change magnitude for runoff. Storage change comparison also showed similar results for the two hotspots in Asia, but not for the central United States. On a global scale, however, a data‐driven study by Borja et al. (2020) found that surface water storage has on average increased over the world during the recent decades, while our model results for the assumed same precipitation in the comparative HI and NHI scenarios indicated human‐driven decreases in water storage going to feed increased evapotranspiration, runoff, and soil moisture (Figure 4). These differences in results indicate possible climate effects, unaccounted for between the scenarios in our study, contributing to the net average water storage increase on global scale reported by Borja et al. (2020).

### 3.2 Impacts of Dams

**Figure 5:**
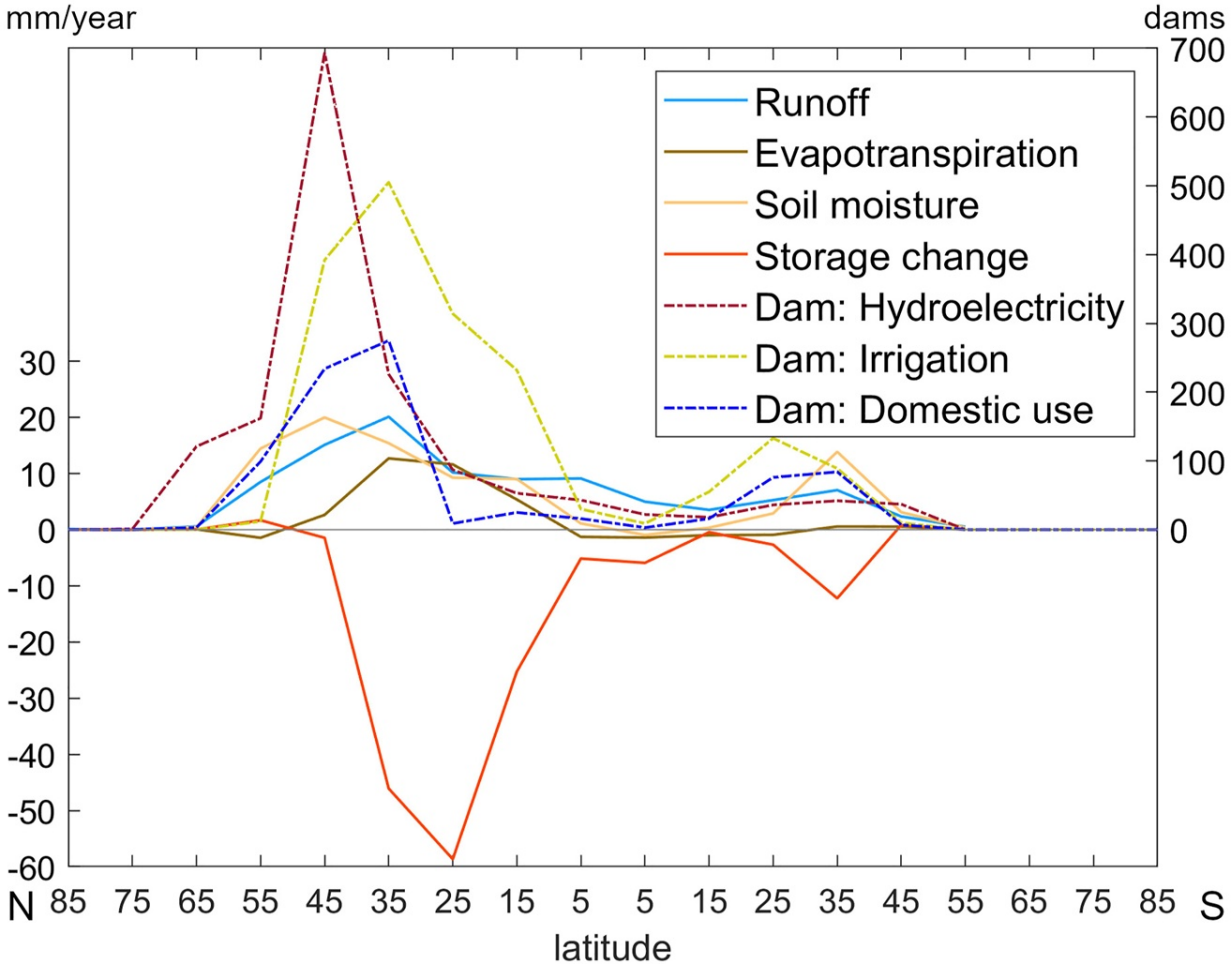
Number of dams of different types and scenario differences in hydrological variables (1st to 99th percentile) at different latitudes.

In addition to population density, world areas with many dams of different types (Figure 1f) coincided to some degree with the areas exhibiting large‐scale hydrological scenario differences (Figure 5). Many dams used primarily for hydroelectric purposes are found in North and South America and Scandinavia (Figure 1f), with high prevalence at northern latitudes with overall higher soil moisture and runoff in the HI than in the NHI scenario (Figure 5). However, scenario differences in evapotranspiration and water storage shifted the direction of change from lower to higher and from higher to lower, respectively, in the middle of this latitudinal band where hydroelectric dams peak, implying overall lower correlation of this than the other types of dams with the greatest scenario differences in hydrology (Figure 5). At lower latitudes, from around 10 to 45°N, peak number of irrigation dams and dams for domestic purposes coincide with the greatest scenario differences in hydrological variables (Figure 5). Moving further south from the peak in the northern hemisphere, hydrological differences between the HI and NHI scenarios decreased to around zero. There are fewer dams in the southern hemisphere, with a peak in number of dams for domestic, irrigation, and hydroelectric purposes around latitudes 25 to 35°S. Among these areas, consisting mostly of part of South America including south Brazil and Uruguay, South Africa and the southern half of Australia, South America has more hydroelectric dams with corresponding increases in storage and a larger increase in runoff. In contrast, South Africa and Australia have almost exclusively irrigation dams and dams for domestic purposes, with the corresponding decrease in storage feeding into increases in runoff and soil moisture.

Several strong correlations were found between hydrological differences between scenarios and number of dams. For example, there were moderate to strong positive correlations with evapotranspiration, and strong to very strong positive correlations with runoff, meaning that evapotranspiration and runoff tended to increase with increasing number of dams. These results are in line with previous findings indicating increased evapotranspiration rates in basins with dams (Destouni et al., 2013), with even greater increases when water is extracted from storage (Moshir Panahi et al., 2020) and when used for irrigation rather than hydropower (Destouni et al., 2013; F. Jaramillo & Destouni, 2015). Increased runoff was also found in Lee and Kim (2017), where two catchments with dams showed yearly increased runoff of around 150 mm resulting from human activities (dams, population changes, and impervious surfaces), as well as in Zhao et al. (2012), although both increased and decreased flows were present when looking at monthly differences compared to pre‐dam conditions. Soil moisture showed a moderate positive correlation with dams in the areas with the greatest global number of irrigation dams (35°N to 45°N), while storage changes showed a very strong negative correlation with dams for all latitudes apart from latitudes 65°N, 75°N, and 45°S which showed a positive correlation with dams used for hydroelectric purposes instead. Dams used for hydroelectric purposes constitute almost all dams in these areas, supporting the tendency that dams use for hydroelectric purposes coincide with areas with increased storage, while other dams coincide with areas with decreased storage.

In summary, when accounting compared with not accounting for human activity, areas worldwide with irrigation dams corresponded well with areas of higher soil moisture and runoff (with 82% and 94%, respectively, of total dams in such areas), while areas with hydroelectric dams corresponded well with areas of higher runoff along with higher storage change (95% and 75%, respectively, of total dams), confirming that hydroelectric dams and irrigation dams show opposite directions of storage change.

### 3.3 Implications

Changes in different hydrological variables differ around the globe, indicating the variations in direct and indirect human impacts on hydrology with geographical location. Human activity and related hydro‐climatic changes, and human responses to these in the form of actions that society takes to adapt to them, are all interconnected and affect in combination hydrological variable changes over time. High‐resolution model results for multiple key water balance variables help fill knowledge gaps for different parts of the world and can provide scientific underpinning for water management planning to ensure sustainable water availability and security. Data availability could cause uncertainties in some parts of the world, with areas with less observational data having larger uncertainties. There is an ongoing need to improve the availability of high‐resolution data for all areas of the world and to assess uncertainties, especially in previously data‐poor regions, to better support sustainable water management and planning. Decreased water flows in rivers and streams have serious implications for water availability and security in the agriculture, industry, energy, and household sectors, with potentially adverse effects on health, the economy, and other societal aspects. For example, in areas with decreased precipitation and increasing agricultural water deficits, the human response may be to build more irrigation dams and/or increase groundwater extraction (depleting groundwater storage; Moshir Panahi et al., 2020). This would enhance agricultural irrigation, with associated evapotranspiration increase, but at the expense of remaining water availability for maintaining surface water runoff and ecosystem health, which may then decline dramatically (Asokan & Destouni, 2014; Destouni & Prieto, 2018; Khazaei et al., 2019).

Increased evapotranspiration in areas with large‐scale irrigation implies human‐driven increase in latent heat flux with associated local‐regional climate cooling (Destouni et al., 2010), that is, an additional human activity effect not accounted for in the NHI scenario modeling with the same climate as in the HI scenario. Analogously, human‐driven decrease in evapotranspiration, as seen from the present scenario simulations for eastern North America, Central America, large parts of central Europe and central South America, sub‐Saharan Africa, and Maritime Southeast Asia, implies increased latent heat flux and associated local‐regional warming, for example, due to urban heat island effects (Corburn, 2009). However, the absence of such effects without human activity was not accounted for in our NHI scenario, which assumed the same climate as in the realistic HI scenario.

Areas identified as most vulnerable and most negatively affected by expected future climate change include areas that lack historical observational data on hydrological changes (Eckstein et al., 2019; Edmonds et al., 2020). In particular, the Intergovernmental Panel on Climate Change (IPCC) has identified Central and West Africa, the Amazon, and Southeast Asia as among the most prominent climate change hotspots around the world (Hewitson et al., 2014). In fact, these regions are among those that have already had high adverse impacts on freshwater ecosystems, and water scarcity problems are increasing in Africa, Asia, the Mediterranean, and on small islands (IPCC, 2022). All areas that showed large decreases in storage in the present study (Figure 3d) are expected to suffer increased severe droughts and increased water stress with future global warming (Byers et al., 2018; IPCC, 2022), with implications for water and food security, especially as many are agricultural areas (Figure 1a). Model‐based studies can improve understanding of human‐driven hydrological changes in such data‐poor regions. In our comparative simulations, Southeast Asia exhibited relatively large scenario differences, indicating considerable human‐driven hydrological impacts, while the Amazon region appeared as relatively unaffected by human activity impacts over the 70‐year simulation period. Central and West Africa also emerged as areas of relatively small human‐driven hydrological impacts in the simulation period, while the impacts were more considerable in Southeast Asia and the southern Mediterranean region, including northern parts of Africa, with relatively high human population density and various intensive human activities (agriculture, irrigation, urbanization with impervious surfaces). Thus, in the future, substantial hydrological change trends can be expected from regional developments involving such activities, and plans for mitigation, adaptation, and/or alternative developments will be needed to safely meet or evade negative expected changes. More research is needed on both impacts and potential mitigation pathways to avoid negative health, economic, societal, and other adverse effects of water deficiency in these most vulnerable and most strongly affected areas.

## 4 Conclusions

This work extended previous model‐based studies of human‐water system interactions through consideration of all four main hydrological variables required for water balance computation, with worldwide geographical coverage that enabled identification of human effect patterns across scales. These included grid‐cell, regional, and global scales, including areas that lack historical observational data for hydrological flows and storage changes. Using the WBM hydrological model, we found human effects on all hydrological variables studied (evapotranspiration, runoff, soil moisture, storage change), but with varying direction and magnitude of change depending on geographical location. As expected, human‐driven hydrological effects were greatest over the northern tropical to temperate zones, where most people live. In these regions, water from considerable storage depletion largely fed increased runoff and evapotranspiration fluxes.

Dam construction emerged as a main factor in regional runoff increase, with dams primarily used for hydroelectric purposes also being associated with water storage increases, while irrigation dams were associated with increased soil moisture. The greatest scenario differences, indicating the greatest human influences on hydrology, were seen in regions that also have many dams for irrigation and domestic purposes, along with high population densities, over the latitudinal band from 10°–45°N. These human activity relationships highlight the need for further research on large‐scale hydrological impacts to assess their importance and, if necessary, overcome biases arising from assuming the same climate under absence of human activity as under its presence. More research is also needed to project future conditions, with particular focus on world regions that may be particularly vulnerable to water availability changes. Southeast Asia and the southern Mediterranean region, including northern parts of Africa, are already experiencing substantial human‐driven hydrological change pressures and may also be particularly negatively impacted by future climate change. This study showed how different key hydrological variables in these and other world regions have likely been affected by human activity developments so far, and the findings can guide further research and management planning for hydrological and water availability trends in future years.

## Data Availability

The water balance model (WBM) is available at https://github.com/wsag/WBM, with input data available at https://dx.doi.org/10.34051/d/2022.2. A full description of the model can be found in Grogan, D.S., Zuidema, S., Prusevich, A., Wollheim, W.M., Glidden, S., and Lammers, R.B. (2022) WBM: A scalable gridded global hydrologic model with water tracking functionality, https://doi.org/10.5194/gmd‐2022‐59.

## References

[eft21120-bib-0001] Asokan, S. M. , & Destouni, G. (2014). Irrigation effects on hydro‐climatic change: Basin‐Wise water balance‐constrained quantification and cross‐regional comparison. Surveys in Geophysics, 35(3), 879–895. 10.1007/s10712-013-9223-5

[eft21120-bib-0002] Bell, B. , Hersbach, H. , Berrisford, P. , Dahlgren, P. , Horánvi, A. , Muñoz Sabater, J. , et al. (2020). ERA5 hourly data on pressure levels from 1950 to 1978 (preliminary version). In Copernicus climate change service (C3S) climate data store (CDS). Retrieved from https://cds.climate.copernicus‐climate.eu/cdsapp#!/dataset/reanalysis‐era5‐pressure‐levels‐preliminary‐back‐extension?tab=overview

[eft21120-bib-0003] Bierkens, M. F. P. (2015). Global hydrology 2015: State, trends, and directions. Water Resources Research, 51(7), 4923–4947. 10.1002/2015WR017173

[eft21120-bib-0004] Bissenbayeva, S. , Abuduwaili, J. , Shokparova, D. , & Saparova, A. (2019). Variation in runoff of the arys river and keles river Watersheds (Kazakhstan), as influenced by climate variation and human activity. Sustainability, 11(17), 4788. 10.3390/su11174788

[eft21120-bib-0005] Borja, S. , Kalantari, Z. , & Destouni, G. (2020). Global Wetting by seasonal surface water over the last decades. Earth's Future, 8(3). 10.1029/2019EF001449

[eft21120-bib-0006] Byers, E. , Gidden, M. , Leclère, D. , Balkovic, J. , Burek, P. , Ebi, K. , et al. (2018). Global exposure and vulnerability to multi‐sector development and climate change hotspots. Environmental Research Letters, 13(5), 055012. 10.1088/1748-9326/aabf45

[eft21120-bib-0007] Corburn, J. (2009). Cities, climate change and urban heat island mitigation: Localising global environmental science. Urban Studies, 46(2), 413–427. 10.1177/0042098008099361

[eft21120-bib-0008] Destouni, G. , Asokan, S. M. , & Jarsjö, J. (2010). Inland hydro‐climatic interaction: Effects of human water use on regional climate: Effects of water use on regional climate. Geophysical Research Letters, 37(18), L18402. 10.1029/2010GL044153

[eft21120-bib-0009] Destouni, G. , Jaramillo, F. , & Prieto, C. (2013). Hydroclimatic shifts driven by human water use for food and energy production. Nature Climate Change, 3(3), 213–217. 10.1038/nclimate1719

[eft21120-bib-0010] Destouni, G. , & Prieto, C. (2018). Robust assessment of uncertain freshwater changes: The case of Greece with large irrigation—And climate‐driven runoff decrease. Water, 10(11), 1645. 10.3390/w10111645

[eft21120-bib-0011] Dey, P. , & Mishra, A. (2017). Separating the impacts of climate change and human activities on streamflow: A review of methodologies and critical assumptions. Journal of Hydrology, 548, 278–290. 10.1016/j.jhydrol.2017.03.014

[eft21120-bib-0012] Döll, P. , Douville, H. , Güntner, A. , Müller Schmied, H. , & Wada, Y. (2016). Modelling freshwater resources at the global scale: Challenges and prospects. Surveys in Geophysics, 37(2), 195–221. 10.1007/s10712-015-9343-1

[eft21120-bib-0013] Eckstein, D. , Künzel, V. , Schäfer, L. , & Winges, M. (2019). Global climate risk index 2020. Germanwatch e.V.

[eft21120-bib-0014] Edmonds, H. K. , Lovell, J. E. , & Lovell, C. A. K. (2020). A new composite climate change vulnerability index. Ecological Indicators, 117, 106529. 10.1016/j.ecolind.2020.106529

[eft21120-bib-0015] Eekhout, J. P. C. , Hunink, J. E. , Terink, W. , & de Vente, J. (2018). Why increased extreme precipitation under climate change negatively affects water security. Hydrology and Earth System Sciences, 22(11), 5935–5946. 10.5194/hess-22-5935-2018

[eft21120-bib-0016] Engström, R. E. , Collste, D. , Cornell, S. E. , Johnson, F. X. , Carlsen, H. , Jaramillo, F. , et al. (2021). Succeeding at home and abroad: Accounting for the international spillovers of cities’ SDG actions. NPJ Urban Sustainability, 1(1), 18. 10.1038/s42949-020-00002-w

[eft21120-bib-0017] Felfelani, F. , Wada, Y. , Longuevergne, L. , & Pokhrel, Y. N. (2017). Natural and human‐induced terrestrial water storage change: A global analysis using hydrological models and GRACE. Journal of Hydrology, 553, 105–118. 10.1016/j.jhydrol.2017.07.048

[eft21120-bib-0018] Fenta, A. A. , Yasuda, H. , Shimizu, K. , & Haregeweyn, N. (2017). Response of streamflow to climate variability and changes in human activities in the semiarid highlands of northern Ethiopia. Regional Environmental Change, 17(4), 1229–1240. 10.1007/s10113-017-1103-y

[eft21120-bib-0019] Ghajarnia, N. , Kalantari, Z. , & Destouni, G. (2021). Data‐driven worldwide quantification of large‐scale hydroclimatic covariation patterns and comparison with Reanalysis and Earth system modeling. Water Resources Research, 57(10), e2020WR029377. 10.1029/2020WR029377

[eft21120-bib-0020] Gordon, L. J. , Steffen, W. , Jonsson, B. F. , Folke, C. , Falkenmark, M. , & Johannessen, A. (2005). Human modification of global water vapor flows from the land surface. Proceedings of the National Academy of Sciences, 102(21), 7612–7617. 10.1073/pnas.0500208102 PMC1140421 15890780

[eft21120-bib-0021] Grogan, D. S. (2016). Global and regional assessments of unsustainable groundwater use in irrigated agriculture (Doctoral Dissertations). University of New Hampshire.

[eft21120-bib-0022] Grogan, D. S. , Wisser, D. , Prusevich, A. , Lammers, R. B. , & Frolking, S. (2017). The use and re‐use of unsustainable groundwater for irrigation: A global budget. Environmental Research Letters, 12(3), 034017. 10.1088/1748-9326/aa5fb2

[eft21120-bib-0023] Grogan, D. S. , Zhang, F. , Prusevich, A. , Lammers, R. B. , Wisser, D. , Glidden, S. , et al. (2015). Quantifying the link between crop production and mined groundwater irrigation in China. Science of the Total Environment, 511, 161–175. 10.1016/j.scitotenv.2014.11.076 25544335

[eft21120-bib-0024] Grogan, D. S. , Zuidema, S. , Prusevich, A. , Wollheim, W. M. , Glidden, S. , & Lammers, R. B. (2022). WBM: A scalable gridded global hydrologic model with water tracking functionality. 10.5194/gmd-2022-59

[eft21120-bib-0025] Gu, J. , Sun, S. , Wang, Y. , Li, X. , Yin, Y. , Sun, J. , & Qi, X. (2021). Sociohydrology: An effective way to reveal the coupled evolution of human and water Systems. Water Resources Management, 35(14), 4995–5010. 10.1007/s11269-021-02984-3

[eft21120-bib-0026] Haddeland, I. , Heinke, J. , Biemans, H. , Eisner, S. , Flörke, M. , Hanasaki, N. , et al. (2013). Global water resources affected by human interventions and climate change. Proceedings of the National Academy of Sciences, 111(9), 3251–3256. 10.1073/pnas.1222475110 PMC3948259 24344275

[eft21120-bib-0027] Hanasaki, N. , Yoshikawa, S. , Pokhrel, Y. , & Kanae, S. (2018). A global hydrological simulation to specify the sources of water used by humans. Hydrology and Earth System Sciences, 22(1), 789–817. 10.5194/hess-22-789-2018

[eft21120-bib-0028] Hersbach, H. , Bell, B. , Berrisford, P. , Biavati, G. , Horánvi, A. , Muñoz Sabater, J. , et al. (2018). ERA5 hourly data on single levels from 1979 to present. Copernicus Climate Change Service (C3S) Climate Data Store (CDS). 10.24381/cds.adbb2d47

[eft21120-bib-0029] Hewitson, B. , Janetos, A. C. , Carter, T. R. , Giorgi, F. , Jones, R. G. , Kwon, W.‐T. , et al. (2014). Regional context. In Barros, V.R. , C. B. Field , D. J. Dokken , M. D. Mastrandrea , K. J. Mach , T. E. Bilir , et al. (Eds.) Climate change 2014: Impacts, adaptation, and vulnerability. Part B: Regional aspects. Contribution of working group II to the fifth assessment report of the intergovernmental Panel on climate change (pp. 1133–1197). Cambridge University Press.

[eft21120-bib-0030] IPCC . (2022). In H. O. Pörtner , D. C. Roberts , M. Tignor , E. S. Poloczanska , K. Mintenbeck , A. Alegría , et al. (Eds.), Climate change 2022: Impacts, adaptation, and vulnerability. Cambridge University Press.

[eft21120-bib-0031] Jaramillo, F. , & Destouni, G. (2014). Developing water change spectra and distinguishing change drivers worldwide. Geophysical Research Letters, 41(23), 8377–8386. 10.1002/2014GL061848

[eft21120-bib-0032] Jaramillo, F. , & Destouni, G. (2015). Local flow regulation and irrigation raise global human water consumption and footprint. Science, 350(6265), 1248–1251. 10.1126/science.aad1010 26785489

[eft21120-bib-0033] Jarsjö, J. , Asokan, S. M. , Prieto, C. , Bring, A. , & Destouni, G. (2012). Hydrological responses to climate change conditioned by historic alterations of land‐use and water‐use. Hydrology and Earth System Sciences, 16(5), 1335–1347. 10.5194/hess-16-1335-2012

[eft21120-bib-0034] Karlsson, J. M. , Jaramillo, F. , & Destouni, G. (2015). Hydro‐climatic and lake change patterns in Arctic permafrost and non‐permafrost areas. Journal of Hydrology, 529, 134–145. 10.1016/j.jhydrol.2015.07.005

[eft21120-bib-0035] Khandu , Forootan, E. , Schumacher, M. , Awange, J. L. , & Müller Schmied, H. (2016). Exploring the influence of precipitation extremes and human water use on total water storage (TWS) changes in the Ganges‐Brahmaputra‐Meghna River Basin. Water Resources Research, 52(3), 2240–2258. 10.1002/2015WR018113

[eft21120-bib-0036] Khazaei, B. , Khatami, S. , Alemohammad, S. H. , Rashidi, L. , Wu, C. , Madani, K. , et al. (2019). Climatic or regionally induced by humans? Tracing hydro‐climatic and land‐use changes to better understand the lake urmia tragedy. Journal of Hydrology, 569, 203–217. 10.1016/j.jhydrol.2018.12.004

[eft21120-bib-0037] Konar, M. , Garcia, M. , Sanderson, M. R. , Yu, D. J. , & Sivapalan, M. (2019). Expanding the scope and foundation of sociohydrology as the science of coupled human‐water systems. Water Resources Research, 55(2), 874–887. 10.1029/2018WR024088

[eft21120-bib-0038] Lee, S. , & Kim, S. (2017). Quantification of hydrological responses due to climate change and human activities over various time scales in South Korea. Water, 9(1), 34. 10.3390/w9010034

[eft21120-bib-0039] Lehner, B. , Liermann, C. R. , Revenga, C. , Vörösmarty, C. , Fekete, B. , Crouzet, P. , et al. (2011). High‐resolution mapping of the world’s reservoirs and dams for sustainable river‐flow management. Frontiers in Ecology and the Environment, 9(9), 494–502. 10.1890/100125

[eft21120-bib-0040] Li, J. , Shi, X. , Chen, Y. D. , & Zhang, L. (2020). Proposing a trend‐based time‐varying approach to assess climate‐ and human‐induced impacts on streamflow. Hydrological Sciences Journal, 65(12), 2043–2056. 10.1080/02626667.2020.1785625

[eft21120-bib-0041] Liu, B. , Zou, X. , Yi, S. , Sneeuw, N. , Cai, J. , & Li, J. (2021). Identifying and separating climate‐ and human‐driven water storage anomalies using GRACE satellite data. Remote Sensing of Environment, 263, 112559. 10.1016/j.rse.2021.112559

[eft21120-bib-0042] Liu, J. , Hertel, T. W. , Lammers, R. B. , Prusevich, A. , Baldos, U. L. C. , Grogan, D. S. , & Frolking, S. (2017). Achieving sustainable irrigation water withdrawals: Global impacts on food security and land use. Environmental Research Letters, 12(10), 104009. 10.1088/1748-9326/aa88db

[eft21120-bib-0043] Liu, J. , Zhou, Z. , Yan, Z. , Gong, J. , Jia, Y. , Xu, C.‐Y. , & Wang, H. (2019). A new approach to separating the impacts of climate change and multiple human activities on water cycle processes based on a distributed hydrological model. Journal of Hydrology, 578, 124096. 10.1016/j.jhydrol.2019.124096

[eft21120-bib-0044] Moshir Panahi, D. , Kalantari, Z. , Ghajarnia, N. , Seifollahi‐Aghmiuni, S. , & Destouni, G. (2020). Variability and change in the hydro‐climate and water resources of Iran over a recent 30‐year period. Scientific Reports, 10(1), 7450. 10.1038/s41598-020-64089-y 32366897 PMC7198531

[eft21120-bib-0045] Müller Schmied, H. , Adam, L. , Eisner, S. , Fink, G. , Flörke, M. , Kim, H. , et al. (2016). Variations of global and continental water balance components as impacted by climate forcing uncertainty and human water use. Hydrology and Earth System Sciences, 20(7), 2877–2898. 10.5194/hess-20-2877-2016

[eft21120-bib-0046] Müller Schmied, H. , Cáceres, D. , Eisner, S. , Flörke, M. , Herbert, C. , Niemann, C. , et al. (2021). The global water resources and use model WaterGAP v2.2d: Model description and evaluation. Geoscientific Model Development, 14(2), 1037–1079. 10.5194/gmd-14-1037-2021

[eft21120-bib-0047] Orth, R. , & Destouni, G. (2018). Drought reduces blue‐water fluxes more strongly than green‐water fluxes in Europe. Nature Communications, 8(1), 3602. 10.1038/s41467-018-06013-7 PMC6127238 30190460

[eft21120-bib-0048] Rakhimova, M. , Liu, T. , Bissenbayeva, S. , Mukanov, Y. , Gafforov, K. S. , Bekpergenova, Z. , & Gulakhmadov, A. (2020). Assessment of the impacts of climate change and human activities on runoff using climate elasticity method and general circulation model (GCM) in the Buqtyrma River Basin, Kazakhstan. Sustainability, 12(12), 4968. 10.3390/su12124968

[eft21120-bib-0049] RGI Consortium . (2017). Randolph glacier inventory – a dataset of global glacier outlines: Version 6.0: Technical report, global land ice measurements from space [Data set]. Digital Media. Retrieved from http://www.glims.org/RGI/randolph60.html

[eft21120-bib-0050] Rost, S. , Gerten, D. , & Heyder, U. (2008). Human alterations of the terrestrial water cycle through land management. Advances in Geosciences, 18, 43–50. 10.5194/adgeo-18-43-2008

[eft21120-bib-0051] Shuster, W. D. , Bonta, J. , Thurston, H. , Warnemuende, E. , & Smith, D. R. (2005). Impacts of impervious surface on watershed hydrology: A review. Urban Water Journal, 2(4), 263–275. 10.1080/15730620500386529

[eft21120-bib-0052] Telteu, C.‐E. , Müller Schmied, H. , Thiery, W. , Leng, G. , Burek, P. , Liu, X. , et al. (2021). Understanding each other’s models: A standard representation of global water models to support improvement, intercomparison, andcommunication. Geoscientific Model Development, 14(6), 3843–3878. 10.5194/gmd-14-3843-2021

[eft21120-bib-0053] Veldkamp, T. I. E. , Wada, Y. , Aerts, J. C. J. H. , Döll, P. , Gosling, S. N. , Liu, J. , et al. (2017). Water scarcity hotspots travel downstream due to human interventions in the 20th and 21st century. Nature Communications, 8(1), 15697. 10.1038/ncomms15697 PMC5481728 28643784

[eft21120-bib-0054] Vörösmarty, C. J. , Green, P. , Salisbury, J. , & Lammers, R. B. (2000). Global water resources: Vulnerability from climate change and population growth. Science, 289(5477), 284–288. 10.1126/science.289.5477.284 10894773

[eft21120-bib-0055] Vörösmarty, C. J. , Moore, B. , Grace, A. L. , Gildea, M. P. , Melillo, J. M. , Peterson, B. J. , et al. (1989). Continental scale models of water balance and fluvial transport: An application to South America. Global Biogeochemical Cycles, 3(3), 241–265. 10.1029/GB003i003p00241

[eft21120-bib-0056] Wada, Y. , Bierkens, M. F. P. , Konar, M. , Liu, J. , Schmied, H. M. , Oki, T. , et al. (2017). Human–water interface in hydrological modelling: Current status and future directions. Hydrology and Earth System Sciences, 25(8), 4169–4193. 10.5194/hess-21-4169-2017

[eft21120-bib-0057] Wada, Y. , de Graaf, I. E. M. , & van Beek, L. P. H. (2016). High‐resolution modeling of human and climate impacts on global water resources. Journal of Advances in Modeling Earth Systems, 8(2), 735–763. 10.1002/2015MS000618

[eft21120-bib-0058] Wisser, D. , Fekete, B. M. , Vorosmarty, C. J. , & Schumann, A. H. (2010). Reconstructing 20th century global hydrography: A contribution to the global terrestrial network‐ hydrology (GTN‐H). Hydrology and Earth System Sciences, 14, 1–24. 10.5194/hess-14-1-2010

[eft21120-bib-0059] Wu, J. , Miao, C. , Zhang, X. , Yang, T. , & Duan, Q. (2017). Detecting the quantitative hydrological response to changes in climate and human activities. Science of the Total Environment, 586, 328–337. 10.1016/j.scitotenv.2017.02.010 28187944

[eft21120-bib-0060] Zaveri, E. , Grogan, D. S. , Fisher‐Vanden, K. , Frolking, S. , Lammers, R. B. , Wrenn, D. H. , et al. (2016). Invisible water, visible impact: Groundwater use and Indian agriculture under climate change. Environmental Research Letters, 11(8), 084005. 10.1088/1748-9326/11/8/084005

[eft21120-bib-0061] Zeng, Y. , Xie, Z. , & Zou, J. (2017). Hydrologic and climatic responses to global anthropogenic groundwater extraction. Journal of Climate, 30(1), 71–90. 10.1175/JCLI-D-16-0209.1

[eft21120-bib-0062] Zhao, Q. H. , Liu, S. L. , Deng, L. , Dong, S. K. , Wang, C. , & Yang, J. J. (2012). Assessing the damming effects on runoff using a multiple linear regression model: A case study of the manwan dam on the lancing river. Procedia Environmental Sciences, 13, 1771–1780. 10.1016/j.proenv.2012.01.171

[eft21120-bib-0063] Zuidema, S. , Grogan, D. , Prusevich, A. , Lammers, R. , Gilmore, S. , & Williams, P. (2020). Interplay of changing irrigation technologies and water reuse: Example from the upper Snake River basin, Idaho, USA. Hydrology and Earth System Sciences, 24(11), 5231–5249. 10.5194/hess-24-5231-2020

